# Microtensile Bond Strength Evaluation of Composite Resin to Discolored Dentin After Amalgam Removal

**DOI:** 10.7759/cureus.7536

**Published:** 2020-04-04

**Authors:** Jyothi Mandava, Sahithi Pamidimukkala, Srujana Karumuri, Ravichandra Ravi, Roopesh Borugadda, Abdul Afraaz

**Affiliations:** 1 Department of Conservative Dentistry and Endodontics, Gandhi Institute of Technology and Management (GITAM) Dental College and Hospital, Visakhapatnam, IND

**Keywords:** amalgam, bond strength, discolored dentin, self-etch adhesive, total-etch adhesive

## Abstract

Background

To obtain prolonged clinical success with composite restorations, better bonding of resin to the tooth substrate is crucial.

Aim

The study was aimed to evaluate the microtensile bond strength (µTBS) of bulk-fill composite resin restorations when bonded to a cavity previously restored with amalgam, comparing with that of freshly prepared dentin.

Materials and method

Mesio-occlusal cavity preparations were done on 80 extracted human mandibular molars with a buccolingual width of 4 mm and a 1.5 mm axial depth by placing the gingival seat 0.5 mm coronal to the cementoenamel junction (CEJ) and were restored with fine-grain amalgam alloy. After thermocycling, the amalgam restorations were removed. Disto-occlusal cavities with similar dimensions of mesial cavities were prepared, and both the proximal surfaces were filled with bulk-fill composite using either etch-and-rinse or self-etch adhesives. Following thermomechanical cyclic loading, all the teeth were sectioned for µTBS testing. Bond strength data expressed in megapascals (MPa) were subjected to statistical analyses using analysis of variance (ANOVA) and Tukey’s multiple post-hoc tests.

Results

The total-etch adhesive exhibited statistically higher bond strength values to both dentin substrates compared to self-etch adhesives (p<0.05). Failure mode analysis reported more of adhesive failures.

Conclusion

The µTBS of bulk-fill composite resin restorations bonded to a cavity previously restored with amalgam was significantly lower than that of freshly prepared dentin. Total-etch adhesives bond strength was higher than self-etch adhesives to both the substrates tested.

## Introduction

The use of composite resin materials through adhesive techniques is becoming a more popular procedure for replacing defective silver amalgam restorations. Achieving a reliable bond between the tooth substrate and composite resin is critical for better retention with minimal microleakage, which, in turn, results in the clinical longevity of the restoration. As dentin is more hydrophilic in nature, it is more difficult to obtain a durable bond of resin to dentin, whereas due to the presence of more inorganic components, it is easier to attain adequate bond with enamel.

Clinically, the dentin available beneath the previous amalgam restoration might have undergone changes like demineralization, remineralization, or sclerosis. Moreover, corrosion being the shortcoming of amalgam, over a period of time, deposits corrosive products at the interface, causing the discoloration of the underlying dentin [1]. This process, in turn, causes changes like reduced mineral volume, increased porosity of intertubular dentin, and degradation of collagen by host-mediated enzymes, all of which inevitably may have a negative impact on the performance of the adhesive bonding. Moreover, the permeability of discolored dentin decreases due to blockage of dentinal tubules with corrosive products, leading to reduced hybrid layer formation and inferior mechanical properties of the adhesive resin restorations [2-4].

The formation of hybridized dentin relies on the dentinal permeability and diffusion capacity of adhesive monomer into the demineralized dentin [5]. Among the two adhesive strategies that are most commonly performed, the etch-and-rinse (ER) procedure is characterized by a complex bonding procedure, whereas self-etching (SE) systems follow a trend towards simplification. Compared to etch-and-rinse, self-etch adhesives simultaneously demineralize and infiltrate, creating homogenous resin infiltration of collagen fibrils, reducing the variation between demineralization depth and infiltration of resin. Studies have reported better stability of the self-etch bonding system due to better protection of collagen fibrils with resin infiltration [6]. However, the efficacy of SE adhesives on enamel demineralization is uncertain. Clinically, very little evidence is available regarding the performance of different adhesives on the discolored dentin substrate.

Bonding stability of adhesive restorations can be evaluated in-vitro by thermo-mechanical cyclic aging procedures. Studies have shown that repetitive contraction, expansion, and mechanical loading stresses accelerate chemical degradation at the tooth-resin interface that can affect bond strength eventually [7-8]. The better a restoration resist the stresses imposed during polymerization and function, the stronger will be the adhesive bond [9].

Hence, this in-vitro study was aimed to investigate and compare the influence of thermo-mechanical stresses on the microtensile bond strength of etch-and-rinse and self-etch adhesives to discolored dentin after amalgam restoration removal. The hypotheses tested were (i) bond strengths of discolored and normal dentin are not dependent on the type of adhesive system used and (ii) thermo-mechanical cyclic aging procedures do not affect bonding effectiveness.

## Materials and methods

The study protocol was approved by the State Health University (D168601021) and ethical clearance was obtained from the institutional review board. Eighty human non-carious mandibular molar teeth having similar dimensions of buccolingual and mesiodistal widths were selected. The sample size was estimated using G power 3.1 software, and 40 samples were indicated as the minimum ideal size required with an alpha error probability of 0.05. Collected teeth after disinfection in chloramine-T (0.5%) solution for two hours were stored in saline at 4°C, maximum for three months before use. The sample mandibular molars were mounted in an auto-polymerizing resin with healthy teeth on each side to maintain the proper proximal contour and contacts. The root surfaces of teeth were covered with polyvinyl siloxane impression material for simulating the periodontal ligament. Teeth were stored at room temperature in distilled water throughout the experimental period.

Restorative procedures

Standardized class II mesio-occlusal cavities were prepared using a # 245 tungsten carbide bur (SS White, New Jersey). A 90° butt joint cavosurface margins were prepared with a 4 mm buccolingual width and 1.5 mm axial wall depth by placing the gingival seat 0.5 mm coronal to the cementoenamel junction (CEJ). After every five tooth preparations, a new bur was used. Following the tooth preparation, two coats of dental varnish (Deepti Dental Products of India Pvt. Ltd., Maharashtra, India) were applied, followed by the placement of the Tofflemire matrix band. Each cavity was then restored with fine-grain dental amalgam alloy (DPI, Maharashtra, India), followed by polishing of the restoration using fine-grit rubber abrasive points.

The amalgam-filled teeth were thermally stressed at 5°C and 55°C (± 1°C) for 10,000 cold and hot cycles with an immersion time of 30 seconds. This procedure was done to generate amalgam corrosion products and to simulate a year of restoration aging [10]. Following the thermocycling procedure, the amalgam restorations were removed from the mesio-occlusal cavities using a # 4 round carbide burs (SS White, New Jersey) in a high-speed handpiece. The cavity preparation was extended to remove a 0.5 mm of discolored dentin under copious air and water coolant, to improve the adhesion of the substrate [11].

A disto-occlusal cavity with similar dimensions of the mesio-occlusal cavity was prepared on all teeth samples using #245 carbide burs (SS White) with a high-speed handpiece. All the internal line angles were rounded. The sample teeth were grouped into two categories (n=40 each) based on the type of bonding procedure, i.e., the etch-and-rinse (ER) or self-etch (SE) adhesive system (Table 1), which were applied to enamel and dentin walls following the manufacturer’s instructions. The Palodent sectional matrix system (Dentsply, New York) was used along with the BiTine separating ring (Dentsply) for restoring mesial and distal class II preparations.

**Table 1:** Materials used in the study

Product name	Composition	Manufacturer	Application
N-etch etching gel.	37 wt.% Phosphoric acid in water, thickeners, and pigments.	Ivoclar Vivadent AG, Schaan, Liechtenstein, Europe.	Applied on enamel for 20 seconds and then on dentin for 15 seconds. Rinsed thoroughly for 10 seconds with water and gently air-dried for 2 seconds.
Tetric N-Bond total-etch adhesive.	Bis-GMA, urethane dimethacrylate, hydroxyethyl methacrylate, phosphonic acid acrylate nano-fillers (Sio_2_), ethanol, initiators, and stabilizers.	Ivoclar Vivadent AG, Schaan, Liechtenstein, Europe.	Applied and gently scrubbed for 10 seconds. A gentle stream of air was used to remove excessive pooling of adhesive and light-cured for 10 seconds.
Tetric N-Bond self-etch adhesive.	Derivatives of bisacrylamide, water, bis-methacrylamide dihydrogen phosphate, amino acid acrylamide, hydroxyalkyl methacrylamide, highly dispersed silicon dioxide, catalysts, and stabilizers.	Ivoclar Vivadent AG, Schaan, Liechtenstein, Europe.	Applied on the enamel and dentin surfaces and scrubbed for 30 seconds with a stream of air. The excess adhesive was removed and light-cured for 10 seconds.
Tetric N-Ceram Bulk-Fill restorative resin.	Urethane dimethacrylate Bisphenol A dimethacrylate, barium glass, ytterbium trifluoride mixed oxide prepolymer.	Ivoclar Vivadent AG, Schaan, Liechtenstein, Europe.	Composite resin was placed in the proximal box first with a maximum of 4 mm increment and light-cured for 20 seconds. Then the occlusal cavity was restored and light cured.

A 4 mm thick increment of Tetric N-Ceram bulk-fill restorative resin (Ivoclar Vivadent AG, Liechtenstein, Europe) was placed in the prepared cavities and polymerized for 20 seconds using a Bluephase C8 light-emitting diode (LED) light-curing unit (Ivoclar Vivadent, Liechtenstein, Austria) with an intensity of 800 mW/cm^2^. Finishing of composite restorations was done with fine-grit Sof-Lex flexible disks (3M ESPE, MN), and polishing was done using rubber cups (Shofu Dental Products, San Marcos) at low speed.

All the composite restored teeth were placed in water at room temperature for 24 hours and were subjected to a thermomechanical loading procedure to simulate clinical performance after the aging process. After thermocycling for 10,000 cycles, the teeth were submitted to 1,00,000 cycles mechanically with an intermittent vertical occlusal loading of 50 Newtons at 20 cycles/minute. The axial force was applied with a 5 mm diameter round piston on the occlusal cuspal inclines at 1 Hz frequency.

Microtensile bond strength evaluation

The teeth were sectioned with a microtome (Leica SP 1600, Germany) using a diamond disc to produce dimensions of 0.9 ± 0.1 mm^2^ thick beam shaped resin-dentin blocks. A total of 160 sections were obtained, i.e., two sections (mesial and distal) from each tooth. The specimens were attached to a custom-made jig with cyanoacrylate glue, and bond strength evaluation was done using a universal testing machine (Autograph, Shimadzu Inc, USA). The tensile load at which the fracture occurred was recorded in units of megapascals. The debonded specimens were examined under a scanning electron microscope (Hitachi, Tokyo, Japan) and the fracture patterns (adhesive, cohesive, or mixed) were assessed by taking photomicrographs at 200X magnification.

Statistical analysis

All the µTBS values recorded in megapascals (MPa) were subjected to statistical analysis using software (SPSS 22.0, IBM, Armonk, NY). A one-way analysis of variance (ANOVA) was used to compare the tensile bond strength among groups. To compare the mean bond strength between two different groups, an independent sample t-test was used. Pair-wise comparisons among the groups were performed by using Tukey multiple post-hoc tests. Statistical analysis was performed at the 95% level of confidence, with the significance level established at P ≤ 0.05.

## Results

The mean bond strength values of total-etch and self-etch adhesives to discolored amalgam replaced dentin were significantly lower (p<0.001) than those to freshly prepared dentin in the same teeth. The etch-and-rinse (ER) adhesives exhibited statistically higher bond strength values to both types of dentin substrates tested as compared to self-etching (SE) adhesives (p<0.001) (Table 2).

**Table 2:** Comparison of mean microtensile bond strength values in MPa by Tukey multiple post-hoc test MPa: megapascal

Groups	Freshly placed composite with etch-and-rinse adhesive	Freshly placed composite with self-etch adhesive	Replaced amalgam with etch-and-rinse adhesive	Replaced amalgam with self-etch adhesive
Mean	43.72	33.56	34.68	23.74
SD	5.37	4.63	4.48	4.31
Freshly placed composite with etch-and-rinse adhesive	-	P<0.001*	P<0.001*	P<0.001*
Freshly placed composite with self-etch adhesive	P<0.001*	-	P=0.7168	P<0.001*
Replaced amalgam with etch-and-rinse adhesive	P<0.001*	P<0.001*	-	P<0.001*
Replaced amalgam with self-etch adhesive	P<0.001*	P<0.001*	P<0.001*	-

**Figure 1:**
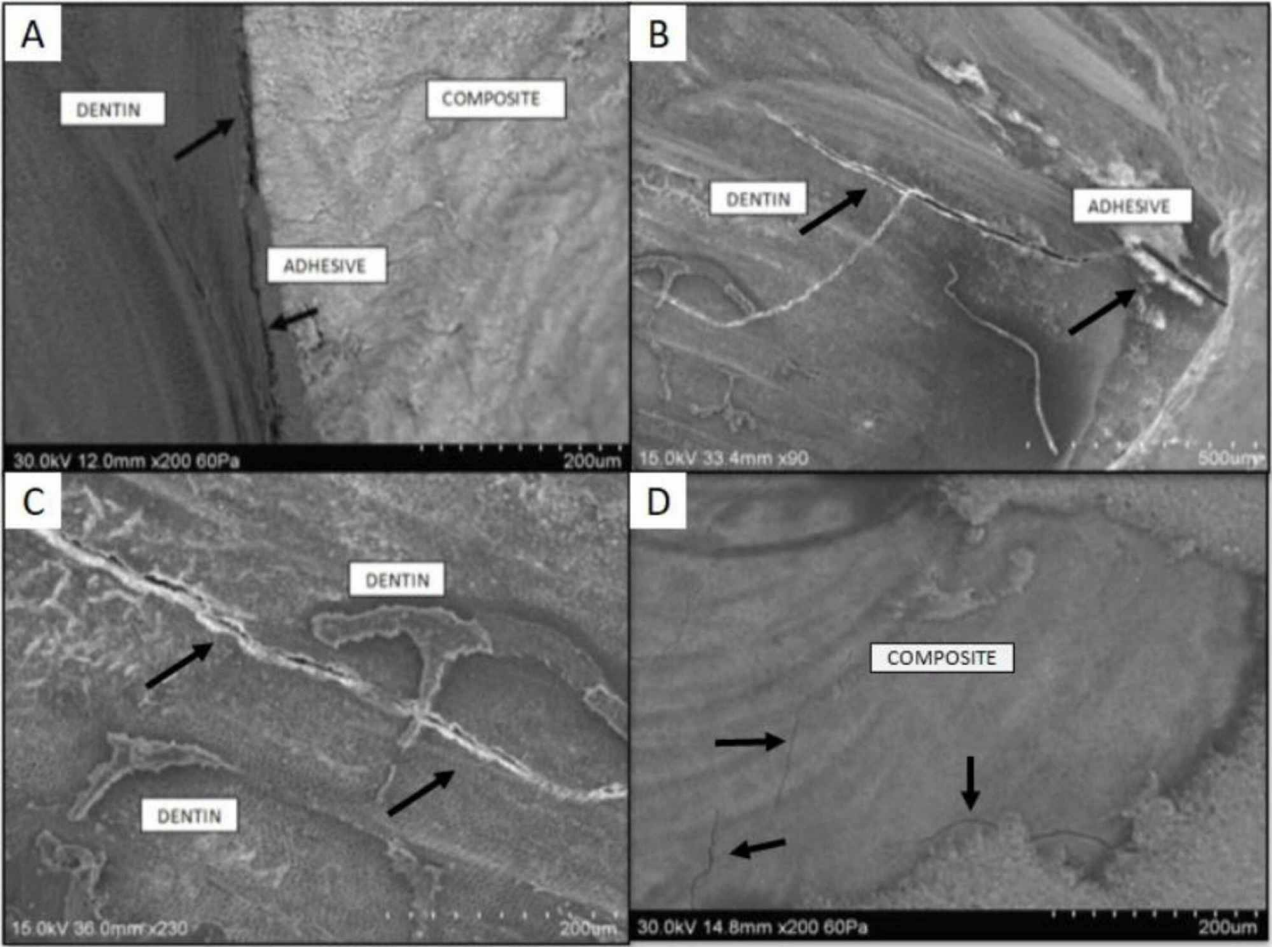
Scanning electron microscopic images of specimens showing failure modes A) Adhesive failure at dentin and composite interface; B) Mixed failure - cohesive failure within the dentin and adhesive failure at the dentin and adhesive interface; C) Cohesive failure within the dentin; D) Cohesive failure within the composite

The microtensile bond strength of freshly cut dentin with SE adhesive and amalgam replaced dentin with ER adhesive were not statistically different (p=0.7168). No significant difference was observed in the mode of failure patterns among the specimens bonded with two different adhesives to different dentin substrates. Overall adhesive failures were more, and mixed failure mode was observed in fewer samples (Figure 1). The highest percentage of adhesive failures were seen in amalgam replaced specimens that were bonded with self-etch adhesive.

## Discussion

Although the microtensile bond strength (µTBS) test is technique sensitive and complicated, being conservative is the major advantage as more specimens can be prepared from a single tooth. Also, we can obtain bond strength values from a relatively small sample, i.e., from less than one mm^2^ cross-sectional area [12-14].

With trituration of the silver alloy and mercury, a matrix structure of Ag_2_Hg_3_ (Ɣ_1_ phase) and Sn_8_Hg (Ɣ_2_ phase) forms around alloy particles Ag_3_Sn (Ɣ phase). The Ɣ_2_ phase is the highly corroded phase and mechanically the weakest phase in conventional amalgams. The Ɣ_2_ phase causes oxidation of Sn and reduces the pH, promoting the acidic environment allowing migration of Sn from amalgam to the dentin restorative interface and getting deposited [2,15]. As the clinician will not be aware of the type of amalgam that was used before while replacing it with composite restoration, to test the worst possible substrate for bonding, conventional amalgam was used in this study.

In an attempt to optimize the infiltration of hydrophobic resins into acid-etched dentin, the ethanol wet bonding technique has been introduced. The research has established that ethanol is the better solvent in the etch-and-rinse primer to produce long-lasting bond strength [16]. The tetric N-bond (two-step) total-etch adhesive was selected in this study for that reason. The tetric N-bond (single step) self-etch adhesive used is a non-rinse adhesive. These adhesives resemble the etch-and-rinse systems by forming a hybrid layer deeper and the dissolved calcium phosphates are embedded within the hybrid layer, without being washed away [17].

Tetric N-Ceram bulk-fill has an increased translucency with an effective depth of cure. In addition to the camphorquinone/amine initiator system, Ivocerin is added for better polymerization. Ivocerin is a germanium-based initiator that has high photocuring activity due to its greater absorption between the 400 and 500 nm wavelengths and forms two free radicals to initiate polymerization [18].

The degradation patterns observed within the hybrid layer are of two types, i.e., loss of resin from interfibrillar spaces and the disorganization of unprotected collagen fibrils [18]. To simulate the intra-oral aging effects, thermo-mechanical cyclic loading was applied to the restored teeth that might correspond to one year of intra-oral functioning [10,14,19].

The results of the present study support the hypothesis that the microtensile bond strengths of both ER and SE adhesives to discolored dentin after the removal of amalgam restorations were lower than that of freshly prepared dentin. This finding was correlating with several previous study results [2,15,20]. It was hypothesized that corrosive amalgam products cause the precipitation of plasma proteins into the dentinal fluid and interfere with smear layer removal, rendering the discolored dentin less etchable than freshly cut dentin. Consequently, the discolored dentin under replaced amalgam restoration is less permeable and reduces the adhesive resin monomer infiltration [15].

Ghavamnasiri et al. recommended a 0.5mm extension of the cavity preparation after the removal of amalgam, to improve the adaptation of composite restorations [11]. They reported similar microleakage values after the removal of discolored dentin beneath the amalgam restoration to that of freshly done composite restorations. In contrast to these findings, Redwan et al. found no difference in microleakage when previously restored amalgam and freshly prepared dentin were tested [1]. The EDX analysis of their study revealed no metal elements in dentin beneath amalgam, and only elements of calcium and phosphorous were identified. Though metal ions did not penetrate the discolored dentin in their study, as compared to freshly prepared dentin, amalgam replaced dentin showed more microleakage, though it was not significant.

The results of the current study revealed lower bond strength values for tetric N self-etch adhesive to both the dentin substrates tested as compared to the total-etch adhesive. With acid-etching, dentin activates and releases matrix metalloproteinases (MMPs), which may partially disintegrate non-resin impregnated collagen within the hybrid layer. In the etch-and-rinse process, phosphoric acid rapidly denatures the MMP activity reducing the gelatinolytic activity. When the pH values reach near zero, whatever gelatinolytic activity is exposed by acid-etching seems to be destroyed [21]. On the other hand, with self-etch adhesives having pH ≥ 2 may activate MMPs at a higher level but may not be acidic enough to denature MMPs [8]. Contrary to this finding, Harnirattisai et al. found a significantly higher bond strength to normal dentin for a Clearfil SE Bond (Kuraray America, Inc., New York) as compared to that of a single bond adhesive [2]. A possible explanation for the higher bond strength to SE adhesive in their study might be due to the fact that thermal/mechanical treatment for the samples was not done. They reported that bond strength for discolored dentin was not statistically different between the two adhesive systems tested.

Lower µTBS for SE adhesives in this study could be attributed to the thinner hybrid layer formation (0.5 to 1 µm) by this adhesive that could not resist the stresses developed during thermal and mechanical cycling [22]. Studies have reported the vulnerability of single-step self-etch adhesives for phase separation due to the droplets or blisters formed at the adhesive-tooth substrate interface. Apart from that, single-step SE adhesives contain a higher amount of water, which is essential for ionization, and that purportedly reduces the conversion rate of the resin [23]. Total-etch adhesive tested in the study have ethanol as a secondary solvent. Ethanol having high solubility and osmotic pressure helps displace water and allow the monomers penetration into the opened dentinal tubules [17]. This might be the reason for attaining higher µTBS values for ER adhesive, as thermo-mechanical stresses will be distributed along the hybrid layer.

The failure mode analysis revealed more of adhesive failures independent of the adhesive system used. This finding is similar to other study results, which reported decreased bond strength with increased adhesive failure after thermocycling [14]. Mitsui et al. reported that with an increased number of thermal/mechanical cycles, the number of mixed failures increases [23]. Due to the partial degradation of resin-dentin interface with aging, mixed failure mode exhibits some cohesive and some adhesive failures [24].

Clinical trials are ideal for the evaluation of dental restorations. An in-vivo study evaluated the longevity of extensive cuspal replacement resin composite restorations that were done upon removal of existing amalgam restorations. They reported that the dentin exposed under earlier amalgam restoration does not impair adhesion of composite resin as they found a good survival rate (96.6%) after 3.5 years with a 0.9% annual failure rate [25].

## Conclusions

The results of this in vitro study provides evidence that etch-and-rinse adhesives provide a higher bond strength to normal dentin and discolored dentin after amalgam removal. Discolored dentin beneath the amalgam restoration decreases the bond strength of an adhesive restoration. Though a single-step SE is desirable in a clinical situation, having acids with hydrophilic and hydrophobic monomers in a single solution might compromise the function of individual constituents. No significant difference was observed in the failure pattern modes among the specimens bonded with two different adhesives to different dentin substrates. Overall adhesive failures are more, and mixed failure mode was observed in a smaller number of samples.
